# Bis{*N*,*N*-bis­[(diphenyl­phosphan­yl)meth­yl]aniline-κ^2^ *P*,*P*′}copper(I) tetra­fluoridoborate

**DOI:** 10.1107/S160053681005186X

**Published:** 2010-12-18

**Authors:** Jing Shang, Li-Li Huang, Ting-Hong Huang, Kai Ma, Qing-Ling Ni

**Affiliations:** a School of Chemistry and Chemical Engineering, Guangxi Normal University, Guilin 541004, People’s Republic of China; b SiChuan College of Chemical Technology, Luzhou 646005, People’s Republic of China

## Abstract

In the cation of the title compound, [Cu(C_32_H_29_NP_2_)_2_]BF_4_, the Cu^I^ atom is four-coordinated in a distorted tetra­hedral geometry by four P atoms from two *N*,*N*-bis­[(diphenyl­phosphan­yl)meth­yl]aniline ligands. In the crystal, the cations are linked by C—H⋯π inter­actions, forming chains along the *a* axis. Intra­molecular C—H⋯N and inter­molecular C—H⋯F hydrogen bonds are also observed.

## Related literature

For the structures and properties of related copper(I) complexes, see: Saravanabharathi *et al.* (2002 ▶); Chen *et al.* (2004 ▶); Sivasankar *et al.* (2004 ▶); Wang *et al.* (2008 ▶); Huang *et al.* (2009 ▶).
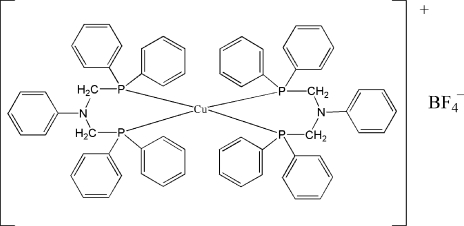


## Experimental

### 

#### Crystal data

[Cu(C_32_H_29_NP_2_)_2_]BF_4_
*M* *_r_* = 1129.35
Triclinic, 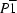

*a* = 11.004 (2) Å
*b* = 12.642 (3) Å
*c* = 21.725 (4) Å
α = 79.601 (3)°
β = 78.593 (3)°
γ = 76.110 (3)°
*V* = 2847.7 (10) Å^3^
*Z* = 2
Mo *K*α radiation
μ = 0.55 mm^−1^
*T* = 296 K
0.20 × 0.10 × 0.10 mm

#### Data collection

Bruker SMART 1000 CCD diffractometer
15689 measured reflections
9918 independent reflections
6522 reflections with *I* > 2σ(*I*)
*R*_int_ = 0.035

#### Refinement

*R*[*F* ^2^ > 2σ(*F* ^2^)] = 0.059
*wR*(*F* ^2^) = 0.198
*S* = 1.06
9918 reflections
685 parameters
H-atom parameters constrained
Δρ_max_ = 0.73 e Å^−3^
Δρ_min_ = −0.47 e Å^−3^

### 

Data collection: *SMART* (Bruker, 1998 ▶); cell refinement: *SAINT* (Bruker, 1998 ▶); data reduction: *SAINT*; program(s) used to solve structure: *SHELXS97* (Sheldrick, 2008 ▶); program(s) used to refine structure: *SHELXL97* (Sheldrick, 2008 ▶); molecular graphics: *SHELXTL* (Sheldrick, 2008 ▶); software used to prepare material for publication: *SHELXTL*.

## Supplementary Material

Crystal structure: contains datablocks I, global. DOI: 10.1107/S160053681005186X/rz2534sup1.cif

Structure factors: contains datablocks I. DOI: 10.1107/S160053681005186X/rz2534Isup2.hkl

Additional supplementary materials:  crystallographic information; 3D view; checkCIF report

## Figures and Tables

**Table 1:** Hydrogen-bond geometry (Å, °) *Cg*1 and *Cg*2 are the centroids of the C33–C38 and C1–C6 rings, respectively.

*D*—H⋯*A*	*D*—H	H⋯*A*	*D*⋯*A*	*D*—H⋯*A*
C22—H22⋯N1	0.93	2.53	3.189 (6)	128
C7—H7*A*⋯F3^i^	0.97	2.25	3.181 (6)	161
C18—H18⋯*Cg*1^i^	0.93	2.72	3.653 (6)	177
C42—H42⋯*Cg*2^ii^	0.93	2.88	3.678 (5)	144

Symmetry codes: (i) 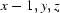; (ii) 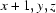.

**Fig. 1.**
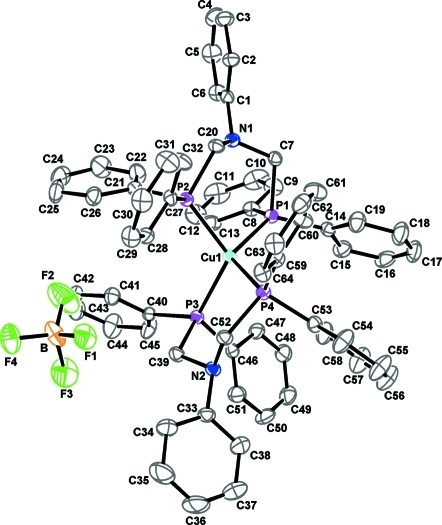
The structure of the title compound with displacement ellipsoids drawn at the 25% probability level. Hydrogen atoms are omitted for clarity.

**Fig. 2.**
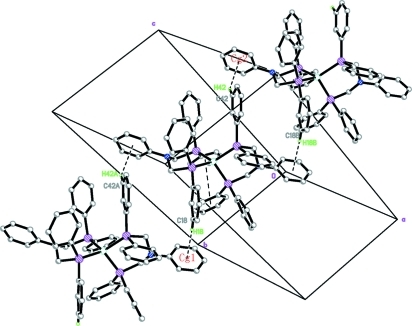
Partial crystal packing of the title compound showing the chains of cations parallel to the a axis. C—H···π interactions are shown as dashed lines.

## supplementary crystallographic information

### Comment

Copper(I) complexes containing phosphine ligands have received much attention so far due to their special structures, novel reactivity, as well as catalytic and luminescent properties (Saravanabharathi *et al.*, 2002; Chen *et al.*,2004; Sivasankar *et al.*, 2004; Wang *et al.*, 2008; Huang *et al.*, 2009). Herein, we report the synthesis and crystal structure of the new mononuclear copper(I) title complex.

In the cation of the title compound, the copper(I) atom adopts a distorted tetrahedral geometry provided by four P atoms from two phosphine ligands (Fig. 1). The Cu—P bond distances are in the range 2.2972 (12)–2.3153 (14) Å. An intramolecular C—H···N hydrogen bond is present (Table 1). In the crystal structure, intermolecular C—H···π interactions link adjacent cations into chains parallel to the *a* axis (Fig. 2). Intermolecular C—H···F hydrogen bonds involving the tetrafluoridoborate anion are also observed (Table 1).

### Experimental

[Cu(CH_3_CN)_4_]BF_4_ (0.0158 g, 0.05 mmol) was added with stirring to a solution of *N*,*N*-bis[(diphenylphosphanyl)methyl]aniline (0.0489 g, 0.10 mmol) in CH_3_CN (5 ml). The resulting solution was allowed to stir for 1 h at room temperature. Slow diffusion of diethyl ether into the solution geve colourless block crystals suitable for X-ray analysis after three days.

### Refinement

Anisotropic displacement parameters were applied to all non-hydrogen atoms. All hydrogen atoms were generated geometrically and refined with a riding model, with C–H = 0.93–0.97 Å, and with *U*_iso_(H) = 1.2 *U*_eq_(C).

### Crystal data

**Table:**

[Cu(C_32_H_29_NP_2_)_2_]BF_4_	*Z* = 2
*M**_r_* = 1129.35	*F*(000) = 1172
Triclinic, *P*1	*D*_x_ = 1.317 Mg m^−^^3^
Hall symbol: -P 1	Mo *K*α radiation, λ = 0.71073 Å
*a* = 11.004 (2) Å	Cell parameters from 1884 reflections
*b* = 12.642 (3) Å	θ = 2.3–21.7°
*c* = 21.725 (4) Å	µ = 0.55 mm^−^^1^
α = 79.601 (3)°	*T* = 296 K
β = 78.593 (3)°	Block, colourless
γ = 76.110 (3)°	0.20 × 0.10 × 0.10 mm
*V* = 2847.7 (10) Å^3^	

### Data collection

**Table:**

Bruker SMART 1000 CCD diffractometer	6522 reflections with *I* > 2σ(*I*)
Radiation source: fine-focus sealed tube	*R*_int_ = 0.035
graphite	θ_max_ = 25.0°, θ_min_ = 1.7°
φ and ω scans	*h* = −13→12
15689 measured reflections	*k* = −14→15
9918 independent reflections	*l* = −25→22

### Refinement

**Table:**

Refinement on *F*^2^	Primary atom site location: structure-invariant direct methods
Least-squares matrix: full	Secondary atom site location: difference Fourier map
*R*[*F*^2^ > 2σ(*F*^2^)] = 0.059	Hydrogen site location: inferred from neighbouring sites
*wR*(*F*^2^) = 0.198	H-atom parameters constrained
*S* = 1.06	*w* = 1/[σ^2^(*F*_o_^2^) + (0.114*P*)^2^] where *P* = (*F*_o_^2^ + 2*F*_c_^2^)/3
9918 reflections	(Δ/σ)_max_ < 0.001
685 parameters	Δρ_max_ = 0.73 e Å^−^^3^
0 restraints	Δρ_min_ = −0.47 e Å^−^^3^

### Special details

**Table:**

Geometry. All e.s.d.'s (except the e.s.d. in the dihedral angle between two l.s. planes) are estimated using the full covariance matrix. The cell e.s.d.'s are taken into account individually in the estimation of e.s.d.'s in distances, angles and torsion angles; correlations between e.s.d.'s in cell parameters are only used when they are defined by crystal symmetry. An approximate (isotropic) treatment of cell e.s.d.'s is used for estimating e.s.d.'s involving l.s. planes.
Refinement. Refinement of *F*^2^ against ALL reflections. The weighted *R*-factor *wR* and goodness of fit *S* are based on *F*^2^, conventional *R*-factors *R* are based on *F*, with *F* set to zero for negative *F*^2^. The threshold expression of *F*^2^ > σ(*F*^2^) is used only for calculating *R*-factors(gt) *etc*. and is not relevant to the choice of reflections for refinement. *R*-factors based on *F*^2^ are statistically about twice as large as those based on *F*, and *R*- factors based on ALL data will be even larger.

### Fractional atomic coordinates and isotropic or equivalent isotropic displacement parameters (Å^2^)

**Table:**

	*x*	*y*	*z*	*U*_iso_*/*U*_eq_	
C1	0.1283 (4)	0.5357 (4)	0.15568 (19)	0.0405 (11)	
C2	0.0669 (5)	0.4537 (4)	0.1860 (2)	0.0449 (12)	
H2	0.0478	0.4436	0.2300	0.054*	
C3	0.0330 (5)	0.3855 (5)	0.1521 (2)	0.0590 (14)	
H3	−0.0078	0.3297	0.1733	0.071*	
C4	0.0596 (6)	0.4004 (5)	0.0871 (3)	0.0689 (17)	
H4	0.0375	0.3543	0.0642	0.083*	
C5	0.1186 (6)	0.4831 (6)	0.0561 (2)	0.0688 (17)	
H5	0.1356	0.4938	0.0120	0.083*	
C6	0.1531 (5)	0.5507 (4)	0.0896 (2)	0.0519 (13)	
H6	0.1931	0.6068	0.0680	0.062*	
C7	0.0813 (4)	0.7133 (4)	0.19308 (18)	0.0377 (10)	
H7A	0.0273	0.7111	0.2342	0.045*	
H7B	0.0275	0.7280	0.1608	0.045*	
C8	0.2195 (5)	0.8354 (4)	0.09759 (18)	0.0450 (12)	
C9	0.1341 (6)	0.8604 (5)	0.0548 (2)	0.0611 (15)	
H9	0.0475	0.8787	0.0695	0.073*	
C10	0.1778 (9)	0.8581 (5)	−0.0101 (3)	0.090 (2)	
H10	0.1208	0.8760	−0.0387	0.108*	
C11	0.3080 (9)	0.8288 (7)	−0.0315 (3)	0.097 (3)	
H11	0.3376	0.8259	−0.0745	0.116*	
C12	0.3903 (7)	0.8051 (6)	0.0092 (3)	0.084 (2)	
H12	0.4767	0.7863	−0.0059	0.100*	
C13	0.3493 (5)	0.8078 (5)	0.0743 (2)	0.0588 (15)	
H13	0.4081	0.7912	0.1019	0.071*	
C14	0.0359 (4)	0.9437 (4)	0.19362 (19)	0.0396 (11)	
C15	0.0258 (6)	1.0410 (5)	0.1515 (3)	0.0627 (15)	
H15	0.0863	1.0475	0.1153	0.075*	
C16	−0.0767 (7)	1.1301 (5)	0.1640 (3)	0.0762 (18)	
H16	−0.0862	1.1944	0.1352	0.091*	
C17	−0.1633 (6)	1.1207 (5)	0.2197 (3)	0.0793 (19)	
H17	−0.2304	1.1794	0.2285	0.095*	
C18	−0.1505 (6)	1.0288 (6)	0.2601 (3)	0.087 (2)	
H18	−0.2085	1.0237	0.2974	0.104*	
C19	−0.0523 (6)	0.9399 (5)	0.2478 (3)	0.0682 (16)	
H19	−0.0459	0.8760	0.2770	0.082*	
C20	0.2100 (4)	0.5532 (4)	0.24857 (19)	0.0406 (11)	
H20A	0.2284	0.4741	0.2492	0.049*	
H20B	0.1418	0.5714	0.2833	0.049*	
C21	0.4793 (5)	0.5208 (4)	0.20502 (19)	0.0407 (11)	
C22	0.4657 (5)	0.5370 (5)	0.1414 (2)	0.0671 (16)	
H22	0.3926	0.5823	0.1284	0.081*	
C23	0.5597 (7)	0.4862 (7)	0.0976 (3)	0.087 (2)	
H23	0.5467	0.4926	0.0559	0.105*	
C24	0.6731 (7)	0.4258 (6)	0.1151 (3)	0.084 (2)	
H24	0.7386	0.3957	0.0849	0.101*	
C25	0.6878 (6)	0.4109 (5)	0.1773 (3)	0.081 (2)	
H25	0.7634	0.3703	0.1898	0.097*	
C26	0.5890 (5)	0.4568 (4)	0.2220 (3)	0.0594 (14)	
H26	0.5985	0.4433	0.2645	0.071*	
C27	0.3773 (4)	0.5157 (4)	0.33799 (19)	0.0398 (11)	
C28	0.4793 (5)	0.5274 (4)	0.3640 (2)	0.0472 (12)	
H28	0.5315	0.5747	0.3423	0.057*	
C29	0.5021 (5)	0.4674 (5)	0.4228 (2)	0.0590 (15)	
H29	0.5712	0.4734	0.4396	0.071*	
C30	0.4250 (6)	0.4008 (5)	0.4553 (2)	0.0636 (16)	
H30	0.4405	0.3626	0.4947	0.076*	
C31	0.3232 (6)	0.3890 (5)	0.4307 (2)	0.0688 (17)	
H31	0.2706	0.3428	0.4534	0.083*	
C32	0.2998 (5)	0.4458 (5)	0.3725 (2)	0.0582 (14)	
H32	0.2315	0.4374	0.3560	0.070*	
C33	0.5601 (5)	0.9522 (4)	0.3714 (2)	0.0475 (12)	
C34	0.6664 (6)	0.8906 (6)	0.3953 (3)	0.0729 (18)	
H34	0.6973	0.8177	0.3885	0.088*	
C35	0.7264 (7)	0.9388 (8)	0.4297 (3)	0.106 (3)	
H35	0.7970	0.8973	0.4469	0.127*	
C36	0.6832 (9)	1.0463 (9)	0.4388 (4)	0.125 (4)	
H36	0.7266	1.0790	0.4601	0.150*	
C37	0.5780 (10)	1.1046 (8)	0.4169 (5)	0.130 (4)	
H37	0.5466	1.1770	0.4245	0.156*	
C38	0.5155 (7)	1.0578 (6)	0.3830 (3)	0.087 (2)	
H38	0.4425	1.0990	0.3680	0.105*	
C39	0.5760 (4)	0.8560 (4)	0.28194 (18)	0.0431 (11)	
H39A	0.6091	0.7788	0.2955	0.052*	
H39B	0.6473	0.8920	0.2682	0.052*	
C40	0.6242 (4)	0.8228 (4)	0.15314 (18)	0.0363 (10)	
C41	0.6929 (5)	0.7163 (4)	0.1626 (2)	0.0537 (13)	
H41	0.6694	0.6696	0.1990	0.064*	
C42	0.7968 (5)	0.6777 (5)	0.1186 (3)	0.0600 (14)	
H42	0.8421	0.6056	0.1257	0.072*	
C43	0.8326 (6)	0.7450 (6)	0.0652 (3)	0.0733 (18)	
H43	0.9036	0.7199	0.0363	0.088*	
C44	0.7635 (7)	0.8495 (6)	0.0546 (3)	0.088 (2)	
H44	0.7864	0.8953	0.0177	0.106*	
C45	0.6589 (6)	0.8883 (5)	0.0986 (2)	0.0667 (16)	
H45	0.6123	0.9598	0.0906	0.080*	
C46	0.4497 (4)	1.0171 (4)	0.18874 (19)	0.0411 (11)	
C47	0.3670 (5)	1.0561 (4)	0.1449 (2)	0.0523 (13)	
H47	0.3365	1.0060	0.1287	0.063*	
C48	0.3294 (6)	1.1660 (5)	0.1248 (3)	0.0652 (15)	
H48	0.2744	1.1895	0.0953	0.078*	
C49	0.3720 (6)	1.2415 (5)	0.1480 (3)	0.0702 (17)	
H49	0.3462	1.3163	0.1344	0.084*	
C50	0.4532 (6)	1.2058 (5)	0.1916 (3)	0.0729 (17)	
H50	0.4823	1.2567	0.2078	0.088*	
C51	0.4924 (5)	1.0938 (5)	0.2117 (2)	0.0593 (14)	
H51	0.5480	1.0705	0.2409	0.071*	
C52	0.4153 (4)	0.8343 (4)	0.37680 (18)	0.0410 (11)	
H52A	0.3931	0.8574	0.4186	0.049*	
H52B	0.4642	0.7593	0.3811	0.049*	
C53	0.1792 (4)	0.9775 (4)	0.35609 (18)	0.0421 (11)	
C54	0.1717 (5)	1.0548 (5)	0.3028 (2)	0.0643 (16)	
H54	0.2039	1.0338	0.2629	0.077*	
C55	0.1158 (7)	1.1645 (6)	0.3087 (3)	0.089 (2)	
H55	0.1134	1.2168	0.2726	0.106*	
C56	0.0644 (7)	1.1962 (5)	0.3667 (3)	0.091 (2)	
H56	0.0290	1.2698	0.3704	0.109*	
C57	0.0656 (7)	1.1180 (5)	0.4196 (3)	0.092 (2)	
H57	0.0268	1.1380	0.4591	0.111*	
C58	0.1241 (6)	1.0102 (5)	0.4143 (2)	0.0743 (19)	
H58	0.1266	0.9584	0.4506	0.089*	
C59	0.1851 (4)	0.7525 (4)	0.41001 (17)	0.0386 (11)	
C60	0.0745 (5)	0.7287 (5)	0.4004 (2)	0.0557 (14)	
H60	0.0452	0.7556	0.3621	0.067*	
C61	0.0068 (6)	0.6656 (5)	0.4471 (2)	0.0658 (16)	
H61	−0.0682	0.6513	0.4403	0.079*	
C62	0.0505 (6)	0.6241 (4)	0.5034 (2)	0.0558 (14)	
H62	0.0047	0.5818	0.5348	0.067*	
C63	0.1599 (5)	0.6443 (4)	0.5136 (2)	0.0544 (14)	
H63	0.1896	0.6145	0.5515	0.065*	
C64	0.2287 (5)	0.7098 (4)	0.4672 (2)	0.0498 (12)	
H64	0.3029	0.7246	0.4747	0.060*	
Cu1	0.32004 (5)	0.78482 (4)	0.24698 (2)	0.03413 (18)	
N1	0.1681 (4)	0.6071 (3)	0.18859 (15)	0.0399 (9)	
N2	0.4930 (3)	0.9051 (3)	0.33536 (15)	0.0393 (9)	
P2	0.35284 (11)	0.59476 (10)	0.26073 (5)	0.0351 (3)	
P1	0.16788 (11)	0.82563 (10)	0.18260 (4)	0.0349 (3)	
P3	0.49074 (11)	0.86914 (10)	0.21416 (4)	0.0348 (3)	
P4	0.26725 (11)	0.83797 (10)	0.34647 (4)	0.0361 (3)	
B	0.8319 (7)	0.5762 (7)	0.3354 (3)	0.071 (2)	
F1	0.7468 (3)	0.5932 (3)	0.38924 (16)	0.0918 (12)	
F2	0.7789 (5)	0.5524 (6)	0.2914 (2)	0.165 (3)	
F3	0.8676 (4)	0.6782 (4)	0.3124 (2)	0.1322 (18)	
F4	0.9401 (4)	0.5048 (4)	0.34690 (16)	0.0974 (13)	

### Atomic displacement parameters (Å^2^)

**Table:**

	*U*^11^	*U*^22^	*U*^33^	*U*^12^	*U*^13^	*U*^23^
C1	0.033 (3)	0.045 (3)	0.047 (2)	−0.004 (2)	−0.015 (2)	−0.012 (2)
C2	0.051 (3)	0.043 (3)	0.047 (2)	−0.015 (2)	−0.013 (2)	−0.0075 (19)
C3	0.064 (4)	0.049 (3)	0.074 (3)	−0.016 (3)	−0.026 (3)	−0.012 (2)
C4	0.081 (5)	0.065 (4)	0.070 (3)	−0.004 (4)	−0.030 (3)	−0.031 (3)
C5	0.075 (4)	0.088 (5)	0.049 (3)	−0.013 (4)	−0.022 (3)	−0.016 (3)
C6	0.060 (4)	0.056 (3)	0.043 (2)	−0.014 (3)	−0.019 (2)	−0.002 (2)
C7	0.035 (3)	0.042 (3)	0.037 (2)	−0.005 (2)	−0.0119 (18)	−0.0056 (17)
C8	0.055 (3)	0.048 (3)	0.035 (2)	−0.017 (2)	−0.011 (2)	−0.0022 (18)
C9	0.071 (4)	0.073 (4)	0.042 (2)	−0.019 (3)	−0.018 (2)	−0.001 (2)
C10	0.156 (8)	0.082 (5)	0.041 (3)	−0.031 (5)	−0.043 (4)	0.004 (3)
C11	0.145 (8)	0.117 (6)	0.034 (3)	−0.056 (6)	0.006 (4)	−0.010 (3)
C12	0.085 (5)	0.109 (6)	0.057 (3)	−0.040 (4)	0.020 (3)	−0.022 (3)
C13	0.062 (4)	0.070 (4)	0.052 (3)	−0.032 (3)	0.002 (3)	−0.015 (2)
C14	0.038 (3)	0.040 (3)	0.042 (2)	−0.009 (2)	−0.0149 (19)	0.0029 (18)
C15	0.071 (4)	0.054 (4)	0.067 (3)	−0.012 (3)	−0.022 (3)	−0.007 (3)
C16	0.095 (5)	0.037 (4)	0.104 (5)	−0.007 (3)	−0.047 (4)	−0.005 (3)
C17	0.064 (4)	0.052 (4)	0.106 (5)	0.010 (3)	−0.002 (4)	−0.013 (3)
C18	0.072 (5)	0.054 (4)	0.115 (5)	0.008 (4)	0.001 (4)	−0.008 (4)
C19	0.068 (4)	0.055 (4)	0.072 (3)	−0.002 (3)	−0.009 (3)	−0.002 (3)
C20	0.042 (3)	0.040 (3)	0.044 (2)	−0.014 (2)	−0.016 (2)	−0.0008 (18)
C21	0.040 (3)	0.035 (3)	0.048 (2)	−0.007 (2)	−0.008 (2)	−0.0074 (18)
C22	0.052 (4)	0.088 (5)	0.061 (3)	−0.003 (3)	−0.011 (3)	−0.024 (3)
C23	0.078 (5)	0.125 (6)	0.064 (3)	−0.022 (5)	0.009 (3)	−0.046 (4)
C24	0.076 (5)	0.081 (5)	0.086 (4)	−0.018 (4)	0.031 (4)	−0.037 (4)
C25	0.058 (4)	0.061 (4)	0.104 (5)	0.013 (3)	0.005 (4)	−0.017 (3)
C26	0.055 (4)	0.046 (3)	0.070 (3)	−0.003 (3)	−0.010 (3)	−0.002 (2)
C27	0.032 (3)	0.040 (3)	0.045 (2)	−0.001 (2)	−0.011 (2)	−0.0049 (18)
C28	0.048 (3)	0.043 (3)	0.053 (3)	−0.008 (2)	−0.016 (2)	−0.008 (2)
C29	0.061 (4)	0.072 (4)	0.044 (2)	−0.003 (3)	−0.022 (2)	−0.008 (2)
C30	0.074 (4)	0.073 (4)	0.037 (2)	−0.005 (3)	−0.019 (3)	0.006 (2)
C31	0.077 (4)	0.071 (4)	0.055 (3)	−0.029 (3)	−0.014 (3)	0.021 (3)
C32	0.058 (4)	0.065 (4)	0.059 (3)	−0.029 (3)	−0.026 (2)	0.013 (2)
C33	0.050 (3)	0.056 (3)	0.045 (2)	−0.017 (3)	−0.009 (2)	−0.018 (2)
C34	0.077 (5)	0.077 (5)	0.079 (4)	−0.016 (4)	−0.028 (3)	−0.030 (3)
C35	0.089 (6)	0.149 (8)	0.101 (5)	−0.029 (5)	−0.042 (4)	−0.042 (5)
C36	0.112 (8)	0.164 (10)	0.139 (7)	−0.046 (7)	−0.021 (6)	−0.103 (7)
C37	0.118 (8)	0.124 (8)	0.179 (9)	−0.019 (6)	−0.029 (7)	−0.108 (7)
C38	0.077 (5)	0.080 (5)	0.119 (5)	−0.002 (4)	−0.031 (4)	−0.053 (4)
C39	0.038 (3)	0.056 (3)	0.040 (2)	−0.010 (2)	−0.0091 (19)	−0.015 (2)
C40	0.033 (3)	0.039 (3)	0.036 (2)	−0.008 (2)	−0.0007 (18)	−0.0099 (17)
C41	0.051 (3)	0.054 (4)	0.053 (3)	−0.008 (3)	−0.004 (2)	−0.008 (2)
C42	0.047 (3)	0.054 (4)	0.078 (3)	0.003 (3)	−0.008 (3)	−0.025 (3)
C43	0.060 (4)	0.085 (5)	0.068 (3)	−0.008 (4)	0.017 (3)	−0.034 (3)
C44	0.087 (5)	0.092 (5)	0.061 (3)	−0.012 (4)	0.032 (3)	−0.004 (3)
C45	0.073 (4)	0.046 (3)	0.061 (3)	0.001 (3)	0.017 (3)	−0.005 (2)
C46	0.033 (3)	0.042 (3)	0.046 (2)	−0.009 (2)	0.003 (2)	−0.0103 (19)
C47	0.056 (3)	0.044 (3)	0.059 (3)	−0.006 (3)	−0.017 (2)	−0.007 (2)
C48	0.059 (4)	0.050 (4)	0.083 (4)	−0.008 (3)	−0.016 (3)	0.000 (3)
C49	0.068 (4)	0.038 (3)	0.095 (4)	−0.009 (3)	0.000 (3)	0.001 (3)
C50	0.088 (5)	0.041 (4)	0.095 (4)	−0.021 (3)	−0.010 (4)	−0.020 (3)
C51	0.063 (4)	0.054 (4)	0.065 (3)	−0.016 (3)	−0.015 (3)	−0.007 (2)
C52	0.041 (3)	0.048 (3)	0.037 (2)	−0.012 (2)	−0.0062 (19)	−0.0108 (19)
C53	0.039 (3)	0.049 (3)	0.036 (2)	−0.008 (2)	−0.0015 (19)	−0.0072 (19)
C54	0.069 (4)	0.063 (4)	0.047 (3)	0.001 (3)	0.007 (2)	−0.009 (2)
C55	0.113 (6)	0.066 (5)	0.065 (3)	0.000 (4)	0.005 (4)	0.000 (3)
C56	0.123 (6)	0.045 (4)	0.090 (4)	0.003 (4)	−0.001 (4)	−0.016 (3)
C57	0.131 (6)	0.062 (5)	0.069 (4)	0.001 (4)	0.011 (4)	−0.031 (3)
C58	0.108 (5)	0.055 (4)	0.047 (3)	−0.003 (4)	0.005 (3)	−0.010 (2)
C59	0.047 (3)	0.036 (3)	0.0306 (19)	−0.006 (2)	−0.0043 (18)	−0.0049 (16)
C60	0.057 (4)	0.071 (4)	0.047 (2)	−0.026 (3)	−0.013 (2)	−0.007 (2)
C61	0.070 (4)	0.075 (4)	0.059 (3)	−0.036 (3)	−0.004 (3)	−0.004 (3)
C62	0.069 (4)	0.043 (3)	0.048 (3)	−0.012 (3)	0.010 (2)	−0.008 (2)
C63	0.059 (4)	0.056 (4)	0.039 (2)	−0.008 (3)	−0.007 (2)	0.008 (2)
C64	0.044 (3)	0.056 (3)	0.047 (2)	−0.008 (3)	−0.008 (2)	−0.005 (2)
Cu1	0.0349 (4)	0.0381 (3)	0.0309 (3)	−0.0084 (3)	−0.0073 (2)	−0.0056 (2)
N1	0.042 (2)	0.036 (2)	0.0456 (18)	−0.0072 (19)	−0.0185 (17)	−0.0045 (15)
N2	0.036 (2)	0.049 (2)	0.0366 (17)	−0.0130 (19)	−0.0030 (15)	−0.0121 (15)
P2	0.0338 (7)	0.0367 (7)	0.0362 (5)	−0.0069 (5)	−0.0101 (5)	−0.0042 (4)
P1	0.0369 (7)	0.0372 (7)	0.0317 (5)	−0.0084 (5)	−0.0096 (4)	−0.0026 (4)
P3	0.0336 (7)	0.0389 (7)	0.0323 (5)	−0.0091 (5)	−0.0029 (4)	−0.0061 (4)
P4	0.0360 (7)	0.0420 (7)	0.0303 (5)	−0.0078 (6)	−0.0041 (4)	−0.0073 (4)
B	0.049 (4)	0.115 (7)	0.040 (3)	−0.018 (4)	−0.010 (3)	0.019 (3)
F1	0.074 (3)	0.105 (3)	0.085 (2)	−0.030 (2)	0.0185 (18)	−0.004 (2)
F2	0.116 (4)	0.293 (8)	0.120 (4)	−0.052 (4)	−0.040 (3)	−0.080 (4)
F3	0.109 (4)	0.118 (4)	0.146 (4)	−0.049 (3)	0.029 (3)	0.022 (3)
F4	0.077 (3)	0.116 (3)	0.076 (2)	0.008 (2)	−0.0128 (19)	0.008 (2)

### Geometric parameters (Å, °)

**Table:**

C1—C2	1.370 (6)	C34—H34	0.9300
C1—C6	1.394 (6)	C35—C36	1.365 (12)
C1—N1	1.438 (5)	C35—H35	0.9300
C2—C3	1.384 (6)	C36—C37	1.338 (11)
C2—H2	0.9300	C36—H36	0.9300
C3—C4	1.372 (7)	C37—C38	1.385 (9)
C3—H3	0.9300	C37—H37	0.9300
C4—C5	1.366 (8)	C38—H38	0.9300
C4—H4	0.9300	C39—N2	1.461 (5)
C5—C6	1.376 (7)	C39—P3	1.860 (4)
C5—H5	0.9300	C39—H39A	0.9700
C6—H6	0.9300	C39—H39B	0.9700
C7—N1	1.456 (5)	C40—C45	1.362 (6)
C7—P1	1.850 (5)	C40—C41	1.378 (7)
C7—H7A	0.9700	C40—P3	1.831 (4)
C7—H7B	0.9700	C41—C42	1.387 (7)
C8—C9	1.395 (7)	C41—H41	0.9300
C8—C13	1.399 (7)	C42—C43	1.359 (8)
C8—P1	1.812 (4)	C42—H42	0.9300
C9—C10	1.400 (7)	C43—C44	1.361 (9)
C9—H9	0.9300	C43—H43	0.9300
C10—C11	1.396 (10)	C44—C45	1.394 (7)
C10—H10	0.9300	C44—H44	0.9300
C11—C12	1.336 (10)	C45—H45	0.9300
C11—H11	0.9300	C46—C51	1.376 (7)
C12—C13	1.400 (7)	C46—C47	1.392 (7)
C12—H12	0.9300	C46—P3	1.825 (5)
C13—H13	0.9300	C47—C48	1.367 (7)
C14—C19	1.371 (7)	C47—H47	0.9300
C14—C15	1.390 (7)	C48—C49	1.366 (8)
C14—P1	1.829 (5)	C48—H48	0.9300
C15—C16	1.411 (8)	C49—C50	1.374 (8)
C15—H15	0.9300	C49—H49	0.9300
C16—C17	1.389 (9)	C50—C51	1.393 (8)
C16—H16	0.9300	C50—H50	0.9300
C17—C18	1.320 (9)	C51—H51	0.9300
C17—H17	0.9300	C52—N2	1.454 (5)
C18—C19	1.384 (8)	C52—P4	1.862 (4)
C18—H18	0.9300	C52—H52A	0.9700
C19—H19	0.9300	C52—H52B	0.9700
C20—N1	1.463 (5)	C53—C54	1.376 (7)
C20—P2	1.851 (4)	C53—C58	1.380 (6)
C20—H20A	0.9700	C53—P4	1.824 (5)
C20—H20B	0.9700	C54—C55	1.393 (8)
C21—C26	1.359 (7)	C54—H54	0.9300
C21—C22	1.392 (6)	C55—C56	1.364 (8)
C21—P2	1.842 (4)	C55—H55	0.9300
C22—C23	1.380 (8)	C56—C57	1.374 (9)
C22—H22	0.9300	C56—H56	0.9300
C23—C24	1.382 (9)	C57—C58	1.375 (8)
C23—H23	0.9300	C57—H57	0.9300
C24—C25	1.366 (9)	C58—H58	0.9300
C24—H24	0.9300	C59—C60	1.383 (7)
C25—C26	1.395 (8)	C59—C64	1.392 (6)
C25—H25	0.9300	C59—P4	1.824 (4)
C26—H26	0.9300	C60—C61	1.384 (7)
C27—C32	1.392 (7)	C60—H60	0.9300
C27—C28	1.401 (6)	C61—C62	1.373 (7)
C27—P2	1.826 (4)	C61—H61	0.9300
C28—C29	1.399 (6)	C62—C63	1.355 (7)
C28—H28	0.9300	C62—H62	0.9300
C29—C30	1.348 (8)	C63—C64	1.404 (7)
C29—H29	0.9300	C63—H63	0.9300
C30—C31	1.382 (8)	C64—H64	0.9300
C30—H30	0.9300	Cu1—P1	2.2972 (12)
C31—C32	1.376 (7)	Cu1—P4	2.3024 (11)
C31—H31	0.9300	Cu1—P3	2.3087 (13)
C32—H32	0.9300	Cu1—P2	2.3153 (14)
C33—C38	1.358 (8)	B—F2	1.324 (8)
C33—C34	1.379 (8)	B—F4	1.347 (8)
C33—N2	1.456 (5)	B—F1	1.360 (7)
C34—C35	1.386 (8)	B—F3	1.415 (9)
			
C2—C1—C6	118.3 (4)	N2—C39—H39B	109.3
C2—C1—N1	123.3 (4)	P3—C39—H39B	109.3
C6—C1—N1	118.3 (4)	H39A—C39—H39B	107.9
C1—C2—C3	121.0 (4)	C45—C40—C41	118.2 (4)
C1—C2—H2	119.5	C45—C40—P3	123.2 (4)
C3—C2—H2	119.5	C41—C40—P3	118.6 (3)
C4—C3—C2	120.0 (5)	C40—C41—C42	121.0 (5)
C4—C3—H3	120.0	C40—C41—H41	119.5
C2—C3—H3	120.0	C42—C41—H41	119.5
C5—C4—C3	119.7 (5)	C43—C42—C41	120.2 (6)
C5—C4—H4	120.1	C43—C42—H42	119.9
C3—C4—H4	120.1	C41—C42—H42	119.9
C4—C5—C6	120.5 (5)	C42—C43—C44	119.3 (5)
C4—C5—H5	119.7	C42—C43—H43	120.3
C6—C5—H5	119.7	C44—C43—H43	120.3
C5—C6—C1	120.4 (5)	C43—C44—C45	120.6 (6)
C5—C6—H6	119.8	C43—C44—H44	119.7
C1—C6—H6	119.8	C45—C44—H44	119.7
N1—C7—P1	111.5 (3)	C40—C45—C44	120.7 (6)
N1—C7—H7A	109.3	C40—C45—H45	119.7
P1—C7—H7A	109.3	C44—C45—H45	119.7
N1—C7—H7B	109.3	C51—C46—C47	117.4 (5)
P1—C7—H7B	109.3	C51—C46—P3	124.1 (4)
H7A—C7—H7B	108.0	C47—C46—P3	118.5 (4)
C9—C8—C13	118.6 (4)	C48—C47—C46	121.8 (5)
C9—C8—P1	122.3 (4)	C48—C47—H47	119.1
C13—C8—P1	118.9 (4)	C46—C47—H47	119.1
C8—C9—C10	120.5 (6)	C49—C48—C47	120.4 (6)
C8—C9—H9	119.7	C49—C48—H48	119.8
C10—C9—H9	119.7	C47—C48—H48	119.8
C11—C10—C9	119.2 (6)	C48—C49—C50	119.3 (6)
C11—C10—H10	120.4	C48—C49—H49	120.3
C9—C10—H10	120.4	C50—C49—H49	120.3
C12—C11—C10	120.5 (5)	C49—C50—C51	120.3 (5)
C12—C11—H11	119.7	C49—C50—H50	119.8
C10—C11—H11	119.7	C51—C50—H50	119.8
C11—C12—C13	121.4 (6)	C46—C51—C50	120.8 (5)
C11—C12—H12	119.3	C46—C51—H51	119.6
C13—C12—H12	119.3	C50—C51—H51	119.6
C8—C13—C12	119.7 (5)	N2—C52—P4	112.5 (3)
C8—C13—H13	120.1	N2—C52—H52A	109.1
C12—C13—H13	120.1	P4—C52—H52A	109.1
C19—C14—C15	118.0 (5)	N2—C52—H52B	109.1
C19—C14—P1	119.1 (4)	P4—C52—H52B	109.1
C15—C14—P1	122.7 (4)	H52A—C52—H52B	107.8
C14—C15—C16	119.7 (5)	C54—C53—C58	118.5 (5)
C14—C15—H15	120.1	C54—C53—P4	118.2 (3)
C16—C15—H15	120.1	C58—C53—P4	123.2 (4)
C17—C16—C15	119.4 (6)	C53—C54—C55	120.0 (5)
C17—C16—H16	120.3	C53—C54—H54	120.0
C15—C16—H16	120.3	C55—C54—H54	120.0
C18—C17—C16	120.2 (6)	C56—C55—C54	120.8 (6)
C18—C17—H17	119.9	C56—C55—H55	119.6
C16—C17—H17	119.9	C54—C55—H55	119.6
C17—C18—C19	121.1 (6)	C55—C56—C57	119.2 (6)
C17—C18—H18	119.5	C55—C56—H56	120.4
C19—C18—H18	119.5	C57—C56—H56	120.4
C14—C19—C18	121.6 (6)	C56—C57—C58	120.2 (5)
C14—C19—H19	119.2	C56—C57—H57	119.9
C18—C19—H19	119.2	C58—C57—H57	119.9
N1—C20—P2	112.4 (3)	C57—C58—C53	121.1 (5)
N1—C20—H20A	109.1	C57—C58—H58	119.4
P2—C20—H20A	109.1	C53—C58—H58	119.4
N1—C20—H20B	109.1	C60—C59—C64	118.7 (4)
P2—C20—H20B	109.1	C60—C59—P4	118.1 (3)
H20A—C20—H20B	107.9	C64—C59—P4	123.2 (4)
C26—C21—C22	118.2 (4)	C59—C60—C61	121.0 (5)
C26—C21—P2	123.3 (4)	C59—C60—H60	119.5
C22—C21—P2	118.4 (4)	C61—C60—H60	119.5
C23—C22—C21	120.5 (6)	C62—C61—C60	119.9 (5)
C23—C22—H22	119.8	C62—C61—H61	120.1
C21—C22—H22	119.8	C60—C61—H61	120.1
C22—C23—C24	120.5 (6)	C63—C62—C61	120.3 (5)
C22—C23—H23	119.8	C63—C62—H62	119.8
C24—C23—H23	119.8	C61—C62—H62	119.8
C25—C24—C23	119.1 (5)	C62—C63—C64	120.6 (5)
C25—C24—H24	120.4	C62—C63—H63	119.7
C23—C24—H24	120.4	C64—C63—H63	119.7
C24—C25—C26	119.9 (6)	C59—C64—C63	119.5 (5)
C24—C25—H25	120.0	C59—C64—H64	120.2
C26—C25—H25	120.0	C63—C64—H64	120.2
C21—C26—C25	121.6 (5)	P1—Cu1—P4	119.06 (5)
C21—C26—H26	119.2	P1—Cu1—P3	117.06 (4)
C25—C26—H26	119.2	P4—Cu1—P3	95.91 (4)
C32—C27—C28	118.6 (4)	P1—Cu1—P2	98.82 (4)
C32—C27—P2	123.8 (4)	P4—Cu1—P2	107.08 (4)
C28—C27—P2	117.6 (4)	P3—Cu1—P2	119.86 (5)
C29—C28—C27	119.4 (5)	C1—N1—C7	114.3 (3)
C29—C28—H28	120.3	C1—N1—C20	113.8 (4)
C27—C28—H28	120.3	C7—N1—C20	113.7 (3)
C30—C29—C28	120.7 (5)	C52—N2—C33	111.4 (3)
C30—C29—H29	119.6	C52—N2—C39	114.9 (4)
C28—C29—H29	119.6	C33—N2—C39	112.7 (3)
C29—C30—C31	120.7 (5)	C27—P2—C21	103.5 (2)
C29—C30—H30	119.7	C27—P2—C20	101.0 (2)
C31—C30—H30	119.7	C21—P2—C20	102.5 (2)
C32—C31—C30	119.8 (5)	C27—P2—Cu1	120.01 (15)
C32—C31—H31	120.1	C21—P2—Cu1	117.57 (15)
C30—C31—H31	120.1	C20—P2—Cu1	109.81 (15)
C31—C32—C27	120.9 (5)	C8—P1—C14	105.0 (2)
C31—C32—H32	119.6	C8—P1—C7	98.7 (2)
C27—C32—H32	119.6	C14—P1—C7	100.9 (2)
C38—C33—C34	119.4 (5)	C8—P1—Cu1	118.19 (17)
C38—C33—N2	119.5 (5)	C14—P1—Cu1	119.29 (14)
C34—C33—N2	121.2 (5)	C7—P1—Cu1	111.60 (14)
C33—C34—C35	119.0 (6)	C46—P3—C40	102.73 (19)
C33—C34—H34	120.5	C46—P3—C39	104.1 (2)
C35—C34—H34	120.5	C40—P3—C39	100.2 (2)
C36—C35—C34	120.8 (7)	C46—P3—Cu1	114.98 (15)
C36—C35—H35	119.6	C40—P3—Cu1	123.12 (15)
C34—C35—H35	119.6	C39—P3—Cu1	109.32 (15)
C37—C36—C35	119.7 (7)	C53—P4—C59	103.5 (2)
C37—C36—H36	120.1	C53—P4—C52	101.4 (2)
C35—C36—H36	120.1	C59—P4—C52	102.6 (2)
C36—C37—C38	120.5 (8)	C53—P4—Cu1	119.58 (13)
C36—C37—H37	119.8	C59—P4—Cu1	118.25 (14)
C38—C37—H37	119.8	C52—P4—Cu1	109.04 (13)
C33—C38—C37	120.6 (7)	F2—B—F4	114.2 (7)
C33—C38—H38	119.7	F2—B—F1	111.4 (6)
C37—C38—H38	119.7	F4—B—F1	112.6 (5)
N2—C39—P3	111.7 (3)	F2—B—F3	107.4 (6)
N2—C39—H39A	109.3	F4—B—F3	105.1 (5)
P3—C39—H39A	109.3	F1—B—F3	105.3 (6)
			
C6—C1—C2—C3	1.6 (7)	C32—C27—P2—C21	−105.7 (4)
N1—C1—C2—C3	−178.6 (5)	C28—C27—P2—C21	75.0 (4)
C1—C2—C3—C4	−0.7 (8)	C32—C27—P2—C20	0.1 (5)
C2—C3—C4—C5	−0.5 (9)	C28—C27—P2—C20	−179.2 (3)
C3—C4—C5—C6	0.9 (9)	C32—C27—P2—Cu1	120.8 (4)
C4—C5—C6—C1	0.0 (9)	C28—C27—P2—Cu1	−58.5 (4)
C2—C1—C6—C5	−1.2 (8)	C26—C21—P2—C27	−24.3 (5)
N1—C1—C6—C5	178.9 (5)	C22—C21—P2—C27	160.1 (4)
C13—C8—C9—C10	−0.2 (8)	C26—C21—P2—C20	−129.0 (4)
P1—C8—C9—C10	−174.6 (4)	C22—C21—P2—C20	55.4 (4)
C8—C9—C10—C11	1.0 (9)	C26—C21—P2—Cu1	110.5 (4)
C9—C10—C11—C12	−1.2 (11)	C22—C21—P2—Cu1	−65.1 (4)
C10—C11—C12—C13	0.5 (11)	N1—C20—P2—C27	179.4 (3)
C9—C8—C13—C12	−0.4 (8)	N1—C20—P2—C21	−73.9 (4)
P1—C8—C13—C12	174.2 (4)	N1—C20—P2—Cu1	51.7 (3)
C11—C12—C13—C8	0.3 (9)	P1—Cu1—P2—C27	−140.72 (17)
C19—C14—C15—C16	−3.0 (8)	P4—Cu1—P2—C27	−16.53 (18)
P1—C14—C15—C16	−178.1 (4)	P3—Cu1—P2—C27	90.97 (18)
C14—C15—C16—C17	2.7 (9)	P1—Cu1—P2—C21	92.04 (17)
C15—C16—C17—C18	−0.9 (11)	P4—Cu1—P2—C21	−143.77 (17)
C16—C17—C18—C19	−0.6 (12)	P3—Cu1—P2—C21	−36.28 (18)
C15—C14—C19—C18	1.5 (9)	P1—Cu1—P2—C20	−24.49 (14)
P1—C14—C19—C18	176.8 (5)	P4—Cu1—P2—C20	99.70 (15)
C17—C18—C19—C14	0.3 (11)	P3—Cu1—P2—C20	−152.80 (14)
C26—C21—C22—C23	2.1 (9)	C9—C8—P1—C14	−43.7 (5)
P2—C21—C22—C23	177.9 (5)	C13—C8—P1—C14	141.9 (4)
C21—C22—C23—C24	−5.3 (10)	C9—C8—P1—C7	60.1 (5)
C22—C23—C24—C25	4.3 (11)	C13—C8—P1—C7	−114.3 (4)
C23—C24—C25—C26	−0.2 (10)	C9—C8—P1—Cu1	−179.6 (4)
C22—C21—C26—C25	2.0 (8)	C13—C8—P1—Cu1	6.0 (5)
P2—C21—C26—C25	−173.6 (5)	C19—C14—P1—C8	156.6 (4)
C24—C25—C26—C21	−3.0 (9)	C15—C14—P1—C8	−28.3 (5)
C32—C27—C28—C29	1.3 (7)	C19—C14—P1—C7	54.4 (5)
P2—C27—C28—C29	−179.3 (4)	C15—C14—P1—C7	−130.5 (4)
C27—C28—C29—C30	−1.8 (8)	C19—C14—P1—Cu1	−68.1 (5)
C28—C29—C30—C31	1.2 (9)	C15—C14—P1—Cu1	107.0 (4)
C29—C30—C31—C32	−0.2 (9)	N1—C7—P1—C8	74.9 (3)
C30—C31—C32—C27	−0.3 (9)	N1—C7—P1—C14	−177.9 (3)
C28—C27—C32—C31	−0.3 (8)	N1—C7—P1—Cu1	−50.2 (3)
P2—C27—C32—C31	−179.6 (4)	P4—Cu1—P1—C8	155.58 (18)
C38—C33—C34—C35	−1.2 (10)	P3—Cu1—P1—C8	40.99 (19)
N2—C33—C34—C35	−179.5 (6)	P2—Cu1—P1—C8	−89.18 (18)
C33—C34—C35—C36	−1.4 (12)	P4—Cu1—P1—C14	26.00 (18)
C34—C35—C36—C37	3.3 (15)	P3—Cu1—P1—C14	−88.59 (17)
C35—C36—C37—C38	−2.5 (16)	P2—Cu1—P1—C14	141.24 (17)
C34—C33—C38—C37	1.9 (11)	P4—Cu1—P1—C7	−91.03 (15)
N2—C33—C38—C37	−179.8 (7)	P3—Cu1—P1—C7	154.38 (14)
C36—C37—C38—C33	−0.1 (15)	P2—Cu1—P1—C7	24.21 (15)
C45—C40—C41—C42	1.7 (8)	C51—C46—P3—C40	−96.1 (4)
P3—C40—C41—C42	−178.4 (4)	C47—C46—P3—C40	86.0 (4)
C40—C41—C42—C43	0.1 (8)	C51—C46—P3—C39	8.0 (4)
C41—C42—C43—C44	−1.7 (10)	C47—C46—P3—C39	−169.9 (4)
C42—C43—C44—C45	1.5 (11)	C51—C46—P3—Cu1	127.6 (4)
C41—C40—C45—C44	−1.9 (9)	C47—C46—P3—Cu1	−50.3 (4)
P3—C40—C45—C44	178.2 (5)	C45—C40—P3—C46	−10.2 (5)
C43—C44—C45—C40	0.3 (11)	C41—C40—P3—C46	169.9 (4)
C51—C46—C47—C48	0.2 (7)	C45—C40—P3—C39	−117.4 (5)
P3—C46—C47—C48	178.3 (4)	C41—C40—P3—C39	62.8 (4)
C46—C47—C48—C49	−0.4 (8)	C45—C40—P3—Cu1	121.4 (4)
C47—C48—C49—C50	0.1 (9)	C41—C40—P3—Cu1	−58.5 (4)
C48—C49—C50—C51	0.4 (9)	N2—C39—P3—C46	64.9 (4)
C47—C46—C51—C50	0.3 (7)	N2—C39—P3—C40	170.9 (3)
P3—C46—C51—C50	−177.7 (4)	N2—C39—P3—Cu1	−58.5 (4)
C49—C50—C51—C46	−0.6 (9)	P1—Cu1—P3—C46	47.73 (15)
C58—C53—C54—C55	3.6 (9)	P4—Cu1—P3—C46	−79.23 (15)
P4—C53—C54—C55	−174.0 (5)	P2—Cu1—P3—C46	167.20 (15)
C53—C54—C55—C56	−2.1 (11)	P1—Cu1—P3—C40	−78.72 (18)
C54—C55—C56—C57	−1.5 (12)	P4—Cu1—P3—C40	154.32 (18)
C55—C56—C57—C58	3.5 (12)	P2—Cu1—P3—C40	40.75 (18)
C56—C57—C58—C53	−2.0 (12)	P1—Cu1—P3—C39	164.39 (16)
C54—C53—C58—C57	−1.7 (9)	P4—Cu1—P3—C39	37.42 (16)
P4—C53—C58—C57	175.8 (5)	P2—Cu1—P3—C39	−76.15 (16)
C64—C59—C60—C61	1.2 (8)	C54—C53—P4—C59	−148.8 (4)
P4—C59—C60—C61	−179.1 (4)	C58—C53—P4—C59	33.8 (5)
C59—C60—C61—C62	−1.1 (9)	C54—C53—P4—C52	105.2 (4)
C60—C61—C62—C63	−0.2 (9)	C58—C53—P4—C52	−72.3 (5)
C61—C62—C63—C64	1.4 (8)	C54—C53—P4—Cu1	−14.7 (5)
C60—C59—C64—C63	−0.1 (7)	C58—C53—P4—Cu1	167.9 (4)
P4—C59—C64—C63	−179.7 (4)	C60—C59—P4—C53	80.7 (4)
C62—C63—C64—C59	−1.2 (8)	C64—C59—P4—C53	−99.7 (4)
C2—C1—N1—C7	−97.9 (5)	C60—C59—P4—C52	−174.1 (4)
C6—C1—N1—C7	82.0 (5)	C64—C59—P4—C52	5.5 (4)
C2—C1—N1—C20	35.1 (6)	C60—C59—P4—Cu1	−54.2 (4)
C6—C1—N1—C20	−145.1 (4)	C64—C59—P4—Cu1	125.5 (3)
P1—C7—N1—C1	−141.0 (3)	N2—C52—P4—C53	−68.8 (3)
P1—C7—N1—C20	86.0 (4)	N2—C52—P4—C59	−175.6 (3)
P2—C20—N1—C1	138.9 (3)	N2—C52—P4—Cu1	58.3 (3)
P2—C20—N1—C7	−87.9 (4)	P1—Cu1—P4—C53	−46.61 (19)
P4—C52—N2—C33	146.8 (3)	P3—Cu1—P4—C53	78.89 (18)
P4—C52—N2—C39	−83.5 (4)	P2—Cu1—P4—C53	−157.37 (18)
C38—C33—N2—C52	−101.4 (6)	P1—Cu1—P4—C59	80.97 (18)
C34—C33—N2—C52	76.9 (6)	P3—Cu1—P4—C59	−153.52 (18)
C38—C33—N2—C39	127.7 (6)	P2—Cu1—P4—C59	−29.78 (18)
C34—C33—N2—C39	−54.0 (6)	P1—Cu1—P4—C52	−162.48 (16)
P3—C39—N2—C52	83.3 (4)	P3—Cu1—P4—C52	−36.97 (17)
P3—C39—N2—C33	−147.6 (3)	P2—Cu1—P4—C52	86.77 (17)

### Hydrogen-bond geometry (Å, °)

**Table:**

Cg1 and Cg2 are the centroids of the C33–C38 and C1–C6 rings, respectively.

**Table:**

*D*—H···*A*	*D*—H	H···*A*	*D*···*A*	*D*—H···*A*
C22—H22···N1	0.93	2.53	3.189 (6)	128
C7—H7A···F3^i^	0.97	2.25	3.181 (6)	161
C18—H18···Cg1^i^	0.93	2.72	3.653 (6)	177
C42—H42···Cg2^ii^	0.93	2.88	3.678 (5)	144

Symmetry codes: (i) *x*−1, *y*, *z*; (ii) *x*+1, *y*, *z*.
